# Characteristics and Related Factors of One-year Transition in Exercise Tolerance Following an Emergency Declaration due to the Coronavirus Disease 2019 Pandemic in Patients on Phase III Cardiac Rehabilitation

**DOI:** 10.1298/ptr.E10232

**Published:** 2023-04-27

**Authors:** Tatsuro KITAYAMA, Taishi TSUJI, Kenta MIKAMI, Naoto USUI, Ryo EMORI, Yasuyuki MARUYAMA, Tadanori HARADA

**Affiliations:** ^1^Department of Rehabilitation, Itabashi Heart Clinic, Japan; ^2^Faculty of Health and Sport Sciences, University of Tsukuba, Japan; ^3^Department of Rehabilitation, Iwatsuki Minami Hospital, Japan; ^4^Department of Rehabilitation, Kisen Hospital, Japan; ^5^Department of Nephrology, Graduate School of Medicine, Juntendo University, Japan; ^6^Department of Rehabilitation, Sonoda Third Hospital, Japan; ^7^Department of Cardiology, Iwatsuki Minami Hospital, Japan; ^8^Department of Cardiology, Itabashi Heart Clinic, Japan

**Keywords:** COVID-19, Peak V̇O_2_, Comorbidity, Gait speed

## Abstract

Objective: This study aimed to understand the long-term transition of exercise tolerance in patients on phase III cardiac rehabilitation (CR) and clarify the characteristics of patients with a high risk of declined exercise tolerance during the first emergency declaration. Methods: Patients who participated in phase III outpatient CR before the first emergency declaration and those who performed cardiopulmonary exercise testing were at ≥2-time points: before and at 3 or 12 months post-emergency declaration. Exercise tolerance transition at 3-time points was analyzed, and whether different social background factors affected the peak oxygen uptake (V̇O_2_) transition method remains to be examined. Results: A total of 101 (median age 74.0 years, 69% men), and both peak V̇O_2_ and anaerobic threshold (AT) significantly declined from pre-declaration to 3 months post-declaration but recovered to levels likely similar from pre-declaration at 12 months (peak V̇O_2_: from 17.3 to 16.7 to 18.7 mL/min/kg; AT: from 11.8 to 11.2 to 11.6 mL/min/kg). Further, patients with multiple comorbidities at pre-declaration had a significantly lower peak V̇O_2_ at 3 months (−1.0 mL/min/kg, p = 0.025) and it remained significantly low in those with a slower gait speed at 12 months after lifting the emergency declaration (−2.5 mL/min/kg, p = 0.009). Conclusion: The emergency declaration declined the exercise tolerance in patients on phase III CR but improved to pre- declaration levels over time, but more likely declined in patients with multiple comorbidities during pre-declaration and those with low-gait speeds were less likely to improve their declined exercise tolerance.

**T**he coronavirus disease 2019 (COVID-19) is affecting the world severely, causing high global morbidity and mortality rates. The World Health Organization declared COVID-19 as a pandemic in March 2020, and the Japanese government declared a state of emergency on April 7, 2020. Consequently, people were forced to restrict their outings and social activities, which significantly stagnated their activities.

A previous study on an older population in Japan reported a 26.5% reduction in physical activity during the emergency declaration^1)^. Another study on patients with heart failure in the Czech Republic also found that the national lockdown for COVID-19 infection control restricted most outdoor activities, except for a few activities, such as shopping for daily necessities, which reduced approximately 1100 steps per day in physical activity^2)^. Furthermore, a previous study on phase III cardiac rehabilitation (CR) patients reported that reduced physical activity time and exercise tolerance were associated with emergency declaration^3)^.

On the other hand, a previous study on community- dwelling elderly people has shown that decreased physical activity with the emergency declaration shows a tendency to improve again^4)^. Furthermore, exercise intolerance due to training interruption has been reported to improve again with training resumption^5)^. Therefore, the emergency declaration is expected to be lifted to improve exercise tolerance again, as it was in the pre-pandemic life, as soon as the epidemic situation improves. However, whether declined exercise tolerance was improved by the emergency declaration and whether the difference between those who improved and unimproved remain unclear. In addition, clarifying the method for screening people at risk of declined exercise tolerance and recommending appropriate physical activities required similar infection control measures due to the emergence of further mutant strains and unknown viruses. It will be valuable knowledge for the development of intervention programs.

Therefore, this study aimed to determine how exercise tolerance in patients on phase III CR changes before the first emergency declaration to 1 year after lifting restrictions and determine the characteristics of patients with low exercise tolerance even after the lifting of the emergency declaration.

## Methods

### Study design and participants

This multicenter retrospective cohort study enrolled patients from four urban medical institutions in Japan (Itabashi Heart Clinic, Iwatsuki Minami Hospital, Kisen Hospital, and Sonoda Third Hospital) who had participated in phase III outpatient CR before the first emergency declaration (from April 7 to May 25, 2020). Patients who underwent a cardiopulmonary exercise test (CPET) approximately 6 months before the emergency declaration (from October 1, 2019 to April 6, 2020) were analyzed. Patients took the CPET test at either or both of the following time points: approximately 3 months (from May 26, 2020 to August 31, 2020) or 12 months (May 26, 2021 to August 31, 2021) after lifting the emergency declaration. The exclusion criteria were as follows: nonindependence in activities of daily living, disagreement to participate, and respiratory exchange ratio (RER) <1.0 in CPET before the emergency declaration^6)^.

This study was conducted in compliance with the “Declaration of Helsinki” and “Ethical Guidelines for Medical Research Involving Human Subjects.” The study protocol was reviewed and approved by the ethical review board of Iwatsuki Minami Hospital (approval no. 33). Consent was obtained from the participants using an opt-out method.

### Evaluation of exercise tolerance and physical function

Participants underwent CPET on a bicycle ergometer in an upright sitting position. A work rate protocol was created using a short-duration ramp test^7)^ and an exercise load electrocardiography program tailored based on physical function. During CPET, expiratory gas analysis was continuously performed through breath-by-breath respiratory gas exchange measurements. Two facilities used the CPEX-1 system (Inter Reha, Tokyo, Japan) and the other two used the Aero Monitor AE-310S (Minato Medical Science, Osaka, Japan) as the breath-by-breath gas analyzers. Peak oxygen uptake (V̇O_2_) was defined as the highest V̇O_2_ value obtained during the last minute of CPET. Anaerobic threshold (AT) was detected using the V-slope method^8)^. CPET was terminated only if the patient requested or if the physician stopped the test for medical reasons, including the occurrence of symptoms or high-risk traits, such as decreased systolic blood pressure of >10 mmHg with increasing workload (persistently below baseline), high-risk ST changes, and sustained ventricular tachycardia^9)^.

Hand-grip strength was measured using a grip strength meter in a standing position. Two measurements were performed on each side, and the maximum value (kg) was used as the representative value. For the gait speed test, the time taken for a usual 4 or 5 m gait was measured twice, the fastest value taken, and m/s calculated.

### Medical record information

The following clinical characteristics were obtained from the patients’ medical records: age, sex, body weight, body mass index (BMI), family structure, employment status, Walk Score as a living environment, disease, comorbidity, β-blocker oral status, and left ventricular ejection fraction.

The number of CR participation was examined in patients during the emergency declaration. The frequency of participating in the outpatient CR program during the emergency declaration period was also investigated. During the first emergency declaration, CR was performed at each facility to achieve infection control measures. The program followed the rehabilitation guidelines for cardiovascular diseases and primarily consisted of warming up, aerobic exercises, resistance training, and cooling down^10)^.

### Statistical analysis

Continuous variables are expressed as mean ± standard deviation when assuming a normal distribution or as median (interquartile range) when not assuming a normal distribution, and the categorical variables are expressed as frequency (%).

Linear mixed-effects models were used to compare exercise tolerance, body weight, and BMI before and 3 months and 12 months after the emergency declaration. The calculated estimated marginal means of peak V̇O_2_, AT, peak RER, body weight, and BMI were used to illustrate changes during measurement. To further confirm the impact of the emergency declaration, a similar analysis was conducted from January 1, 2020 to April 6, 2020, only for participants who took the CPET prior to the emergency declaration.

Then, an unpaired *t*-test was performed to compare the peak V̇O_2_ before the emergency declaration in the presence and absence of background factors. The presence of a background factor is defined as 1, whereas the absence of a background factor is defined as 0. Gender was defined as 1 for men and 0 for women, household composition as 1 for those living alone and 0 for those living together, and the employment status as 1 for workers and 0 for nonworkers. The presence or absence of multiple comorbidities was defined as having three or more or two or fewer comorbidities, respectively, with 1 having three or more comorbidities and 0 having two or fewer comorbidities^11)^. For the living environment, the Walk Score, an index used for evaluating neighborhood walkability, is defined as a low value for <90 points, with 1 for <90 points and 0 for ≥90 points^12^^,^^13)^. Grip strength and usual gait speed were used as physical functions at pre-emergency declaration. According to the revised Japanese version of the Cardiovascular Health Study (J-CHS) criteria, grip strength is defined as low grip strength of <28 kg for men and <18 kg for women, with normal grip strength being higher. The usual gait speed is defined as a slow gait speed when showing <1.0 m/s and as a normal gait speed when showing >1.0 m/s^14)^. The low grip strength group was set to 1, the normal grip strength group was set to 0, the slow gait speed group was set to 1, and the normal gait speed group was set to 0.

Next, background factors were found to be associated with peak V̇O_2_ changes from the pre-emergency declaration to 3 months and 12 months after lifting. A linear mixed-effects model was used, with an objective variable as peak V̇O_2_ and background factors (sex, household composition, employment status, comorbidity, living environment, grip strength, and usual gait speed), and the interaction terms during measurement as explanatory variables separately. All background factors were used before the pre-emergency declaration. Adjustment variables were age, sex (except when sex was entered in the interaction term), and peak V̇O_2_ pre-emergency declaration. As a result, this generated point estimates (unstandardized coefficients) and 95% confidence intervals for the difference at 3 and 12 months after lifting the emergency declaration for those with a background factor of 1 versus those without a background factor of 0. Further, using the estimated marginal mean, the transition during measurement of the peak V̇O_2_ with and without background factor is illustrated.

The significance level was set to *p* <0.05 in all statistical analyses. The Stata/BE 17.0 (College Station, TX, USA) was used for statistical analysis.

## Results

Among the 119 enrolled participants, 18 were excluded (3 who needed assistance with activities of daily living, 1 who refused to participate, and 14 with an inadequate load on CPET). Finally, the data of 101 people were analyzed. A total of 38 participants were able to perform CPET at both 3 and 12 months after the emergency declaration was lifted, 90 were able to perform CPET after 3 months, and 11 were able to perform CPET after 12 months. The median age of the participants was 74.0 (67.0–80.0 years), and 69 (69%) were men. Participants’ primary disease for CR was myocardial infarction in 25 (24.8%), angina pectoris in 22 (21.8%), cardiac surgery in 13 (12.9%), chronic heart failure in 33 (32.7%), aortic disease in 1 (1.0%), peripheral artery disease in 3 (3.0%), and transcatheter aortic valve implantation in 4 (4.0%). The median left ventricular ejection fraction of participants was 60.0% (49.0%–64.0%), and 75 (74.3%) of them were taking β-blockers. Furthermore, 62 (61.4%) had three or more comorbidities, 32 (31.7%) were working, and 25 (24.8%) were living alone. Participants had a median Walk Score of 80.0 (67.0–88.0), and the median number of patients who participated in the outpatient CR during the emergency declaration was 4 (2.0–6.0) (Table 1).

**Table 1.** Clinical characteristics

	Overall(*n* = 101)
Age, years	74.0 (67.0–80.0)
Men, *n* (%)	69.0 (68.3)
Body weight (kg)	60.4 ± 11.3
BMI	23.4 (20.8–25.4)
Disease, *n* (%)	
Myocardial infarction	25 (24.8)
Angina pectoris	22 (21.8)
Postcardiac surgery	13 (12.9)
Chronic heart failure	33 (32.7)
Aortic disease	1 (1.0)
Peripheral artery disease	3 (3.0)
Post TAVI	4 (4.0)
Comorbidity, *n* (%)	
Hypertension	85 (84.1)
Dyslipidemia	71 (70.3)
Former or current smoker	56 (55.4)
Diabetes mellitus	41 (41.0)
Cardiovascular disease	33 (32.7)
Chronic kidney disease	26 (25.7)
Orthopedic disorders	25 (24.8)
Malignant tumor	11 (10.9)
Cerebrovascular disease	7 (6.9)
Respiratory disease	2 (2.0)
LVEF, %	60.0 (49.0–64.0)
β-blocker medication, *n* (%)	75 (74.3)
Multiple comorbidities 3 or more, *n* (%)	62 (61.4)
Worker, *n* (%)	32 (31.7)
Living alone, *n* (%)	25 (24.8)
Walk Score, points	80.0 (67.0–88.0)
Number of CR during the emergency declaration, times	4.0 (2.0–6.0)

Continuous variables are expressed as mean with standard deviation in parenthesis or median with interquartile range in parenthesis BMI, body mass index; TAVI, transcatheter aortic valve implantation; LVEF, left ventricular ejection fraction; CR, cardiac rehabilitation

**Fig. 1.**
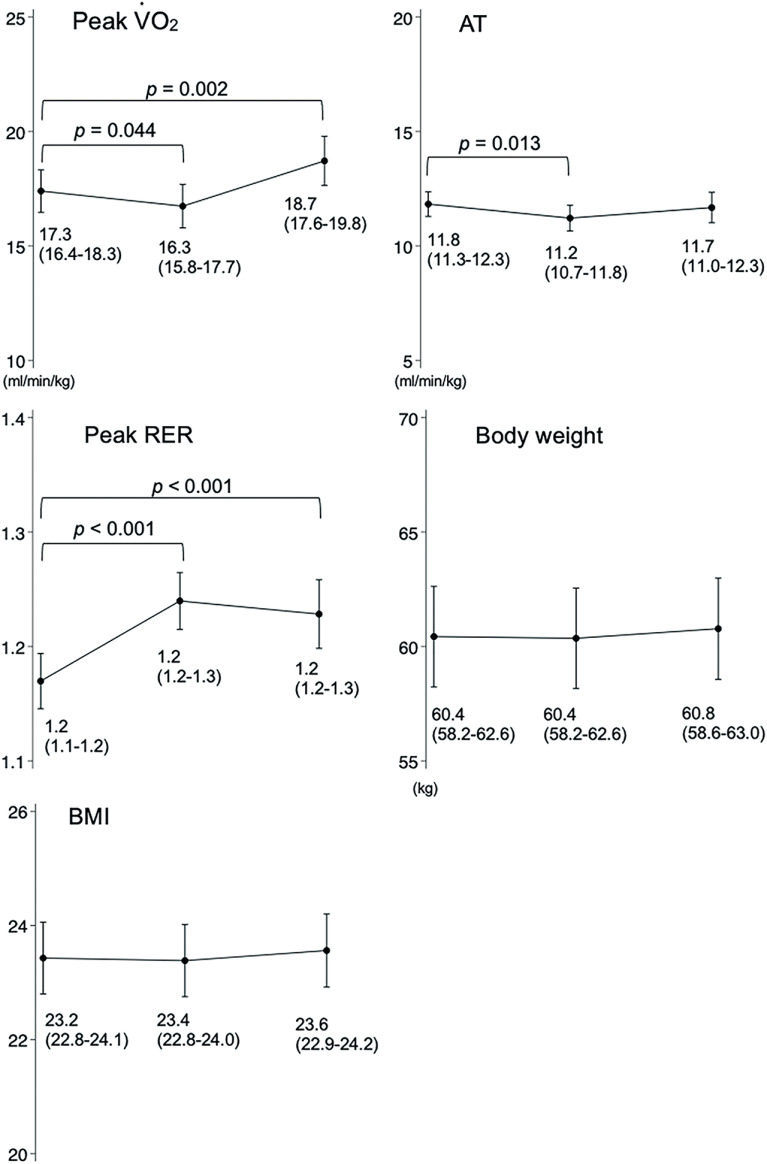
Exercise tolerance and body composition before the emergency declaration, 3 months after lifting, and 12 months after lifting

Figure 1 shows changes in the peak V̇O_2_, AT, peak RER, body weight, and BMI at pre-emergency declaration, 3 months after lifting, and 12 months after lifting. The peak V̇O_2_ significantly decreased from 17.3 mL/min/kg to 16.3 mL/min/kg at 3 months (*p* = 0.044) and to 18.7 mL/min/kg at 12 months after lifting the emergency declaration, a significant increase compared to that in pre-emergency declaration (*p* = 0.002). The AT significantly decreased from 11.8 mL/min/kg to 11.2 mL/min/kg at 3 months after lifting the emergency declaration (*p* = 0.013) compared to pre-emergency declaration. Notably, body weight and BMI did not change. There were 56 participants who performed CPET prior to the emergency declaration from January 1, 2020 to April 6, 2020. The trends in peak V̇O_2_ and AT were compared to the analysis with 101 participants and the trends were the same (Appendices 1 and 2; all supplementary files are available online).

Values are, in order, estimated marginal mean values before the emergency declaration, 3 months after lifting the emergency declaration, and 12 months after lifting the emergency declaration.

V̇O_2_, oxygen uptake; AT, anaerobic threshold; RER, respiratory exchange ratio; BMI, body mass index

**Fig. 2.**
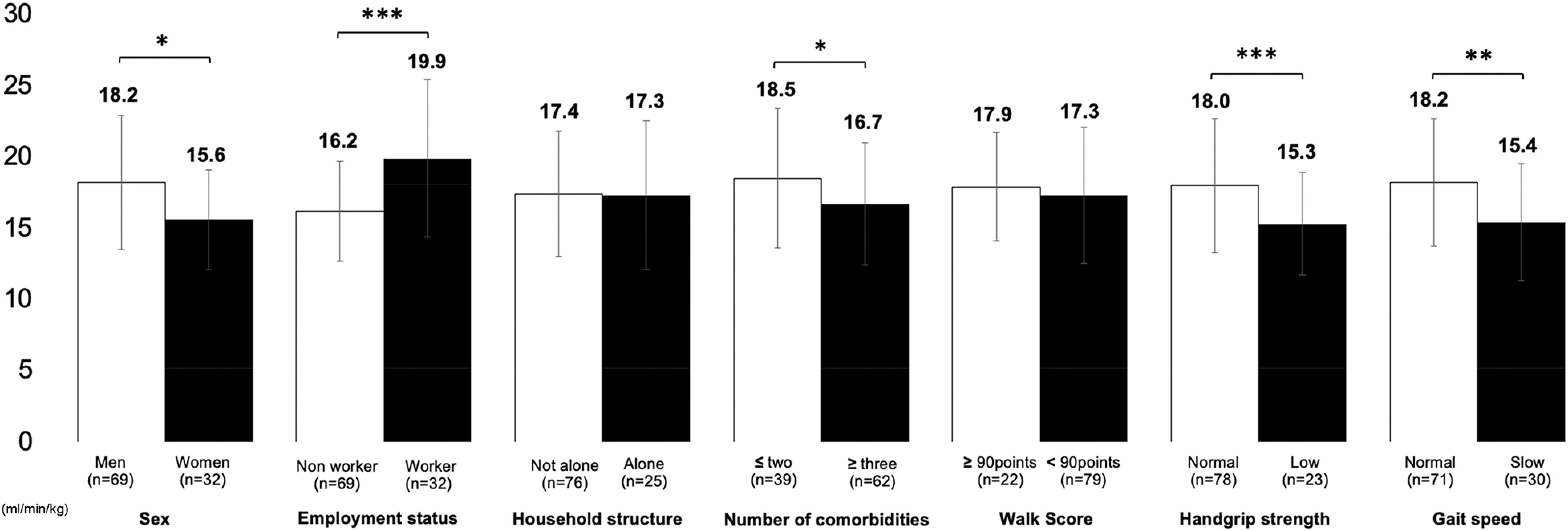
Peak V̇O_2_ before the emergency declaration classified by background

Figure 2 shows the peak V̇O_2_ during the pre-emergency declaration for each background factor. Comparing men with women (15.6 ± 4.7 vs. 18.2 ± 4.7 mL/min/kg, *p* = 0.006) and workers with nonworkers (16.2 ± 3.5 vs. 19.9 ± 5.4 mL/min, *p* <0.001), the peak V̇O_2_ was high, and a statistically significant difference was observed. Furthermore, when comparing patients with low grip strength and those with normal grip strength (18.0 ± 4.6 vs. 15.3 ± 3.5 mL/min/kg, *p* = 0.012), those with slow gait speed and those normal (18.2 ± 4.5 vs. 15.4 ± 4.0 mL/min/kg, *p* = 0.005), and those who had three or more comorbidities (18.5 ± 4.8 vs. 16.6 ± 4.2 mL/min/kg, *p* = 0.049) and those who had two or fewer comorbidities, the peak V̇O_2_ was low, and a statistically significant difference was observed.

Error bars indicate 95% CI.

*: *p* <0.05, **: *p* <0.01, ***: *p* <0.001

V̇O_2_, oxygen uptake; CI, confidence interval

**Fig. 3.**
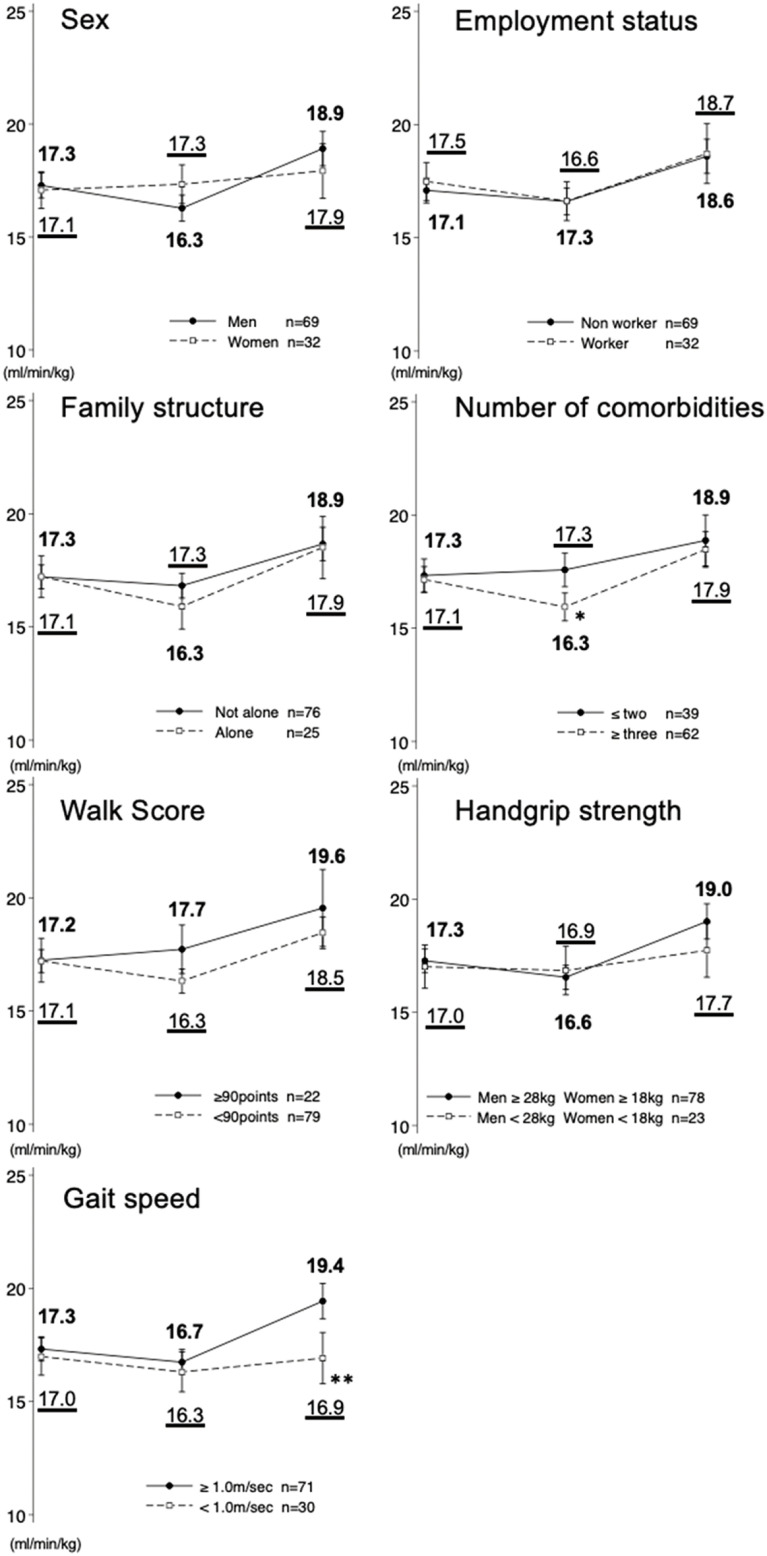
Peak V̇O_2_ background at each evaluation point

Table 2 shows the unstandardized coefficients, 95% confidence intervals, and *p*-values calculated by imputing the interaction term between the presence and absence of background factors × time of evaluation to the linear mixed-effects model. Moreover, the estimated marginal mean based on the results is shown in Figure 3. At 3 months after the lifting of the emergency declaration, based on the values before the emergency declaration, those with ≥3 comorbidities had significantly lower peak V̇O_2_ than those ≤2 (−1.46 mL/min/kg, *p* = 0.025). At 12 months after lifting the emergency declaration, patients with a slow gait speed before the emergency declaration had a significantly lower peak V̇O_2_ than those with a normal gait speed (−2.18 mL/min/kg, *p* = 0.009).

**Table 2.** Results of the interaction between peak V̇O_2_ transition and background factors × evaluation period in the linear mixed- effects model

Background factor	Evaluation period*	Coefficient Background factor × Evaluation period	95% CI	*p*
Men	3 months after lifting	–1.27	–2.62, 0.08	0.066
	12 months after lifting	0.78	–0.89, 2.45	0.361
Worker	3 months after lifting	–0.38	–1.74, 0.99	0.588
	12 months after lifting	–0.27	–2.04, 1.49	0.761
Living alone	3 months after lifting	–0.93	–2.42, 0.56	0.223
	12 months after lifting	–0.16	–1.99, 1.67	0.863
Number of comorbidities (≥3)	3 months after lifting	–1.46	–2.73, –0.18	0.025
	12 months after lifting	–0.22	–1.82, 1.39	0.788
Walk Score (<90 points)	3 months after lifting	–1.36	–2.92, 0.20	0.087
	12 months after lifting	–1.05	–3.16, 1.05	0.326
Low handgrip strength (men <28 kg, women <18 kg)	3 months after lifting	0.56	–0.99, 2.21	0.479
	12 months after lifting	–1.01	–2.73, 0.71	0.248
Slow gait speed (<1.0 m/s)	3 months after lifting	–0.10	–1.47, 1.28	0.891
	12 months after lifting	−2.18	−3.81, −0.55	0.009

*Estimated the difference of changes in the evaluation period with background factor (1) against no background factor (0), based on the period before the emergency declaration

V̇O_2_, oxygen uptake; CI, confidence interval

Values are, in order, estimated marginal mean values before the emergency declaration, 3 months after lifting the emergency declaration, and 12 months after lifting the declaration.

Error bars indicate 95% CI.

The results show that there were significant differences between the groups over time, based on the time before the emergency declaration.

*: *p* <0.05, **: *p* <0.01

V̇O_2_, oxygen uptake; CI, confidence interval

## Discussion

This is the first study to verify the transition of exercise tolerance and related factors in patients on phase III CR from before to 12 months after lifting the emergency declaration. The results showed that both the peak V̇O_2_ and AT decreased in the overall participants 3 months after lifting the emergency declaration but improved to the level before the emergency declaration and continued till 12 months after lifting the emergency declaration. The peak V̇O_2_ transition was significantly lower in participants with ≥3 comorbidities than in those with ≤2 comorbidities at 3 months after lifting. At 12 months after lifting the emergency declaration, those with slower gait speeds before the emergency declaration were less likely to improve their peak V̇O_2_ than those with normal gait speeds.

Previous studies in patients on phase III CR have reported that physical activity was positively correlated with the peak V̇O_2_^15)^ and that a decreased physical activity time during the emergency declaration period was associated with decreased exercise tolerance^3)^. Conversely, a previous study of patients with chronic heart failure reported that physical function and frailty worsened before and after interrupting the outpatient CR due to emergency declaration but improved again when outpatient CR was resumed^16)^. Furthermore, a previous study of elderly community-dwelling people reported that the amount of physical activity declined after the emergency declaration showing a tendency to improve again from 3 months after lifting the emergency declaration^4)^. Generally, exercise intolerance due to training interruption improves again when training is resumed^5)^. Considering these previous studies, the frequency of participation in outpatient CR and the amount of physical activity recovered in study participants after lifting the emergency declaration improved. As a result, exercise tolerance improved again.

The presence of ≥3 comorbidities increased the risk of declined exercise tolerance for 3 months after lifting the emergency declaration. A previous study has reported that patients with heart disease and lifestyle-related diseases are at a high risk of serious injury due to COVID-19^17)^. Furthermore, Japanese information media has reported the risk of severe COVID-19 infection to be higher in patients with underlying diseases. These factors may have influenced those with more comorbidities to refrain excessively from going out and engaging in activities. As a result, the amount of physical activity is more likely to decrease, which may have led to a decreased peak V̇O_2_.

Previous studies on community-dwelling elderly people have reported that grip strength and gait speed are both predictors of peak V̇O_2_^18)^. However, the results of the present study suggested that people with a slow gait speed before the emergency declaration had a high risk of a declined peak V̇O_2_ 12 months after lifting the emergency declaration. Previous studies have shown that gait speed predicts the subsequent activities of daily living decline^19)^ and that those with a gait speed of <1.0 m/s have difficulty walking at crosswalks^20)^; therefore, gait speed can have a direct impact on daily life. Furthermore, the study participants lived in the metropolitan area, which tended to have a particularly poor COVID-19 infection rate in Japan^21)^, and were asked to refrain from leaving the home unnecessarily for a long time even after lifting of the first emergency declaration. Therefore, slow gait speed may have limited the amount of activity for a longer time, resulting in lesser improved peak V̇O_2_. Thus, it may be necessary to recommend more aggressive self-training to maintain activity levels in those with slower gait speed.

This study has the strengths of using longitudinal data to investigate exercise tolerance in patients on phase III CR before and after the emergency declaration due to the COVID-19 epidemic. Conversely, some limitations should still be considered. First, the amount of physical activity during the study period is inadequately investigated. Therefore, whether the study results reduce the physical activity associated with the emergency declaration or normal aging- related changes remains controversial. Second, although the study was conducted in a multicenter setting, the number of patients was less. Third, patients usually participated in the outpatient CR and performed CPET. Therefore, it is possible that those with relatively good physical function and a high motivation to exercise are included in the population. Therefore, physical function was relatively maintained and was biased toward those who were highly motivated to exercise. Sampling biases may have occurred and influenced the results. Fourth, although we have described background factors that make peak V̇O_2_ more likely to decline and less likely to improve, these subjects are generally a difficult population to recover from, and it is not possible to confirm whether the differences observed are the result of the emergency declaration or not.

## Conclusion

Before and after the first emergency declaration, exercise tolerance of patients on phase III CR declined; however, after 1 year, it improved to the level as before the declaration. However, those with ≥3 comorbidities before the declaration declined to 3 months after lifting and those with a gait speed of <1.0 m/s had low peak V̇O_2_ until 12 months after lifting the emergency declaration. This study suggests that increasing physical activity and providing an intervention program for patients with such characteristics should be promoted.

## Acknowledgments

We would like to express our sincere gratitude to the coauthors who provided valuable data for this study.

## Conflict of Interest

The authors have no conflicts of interest to declare.

## Supplementary Material (Appendices)

Appendix 1. Clinical characteristics in 56 participants

Appendix 2. Exercise tolerance and body composition of 56 participants before the emergency declaration, 3 months after lifting, and 12 months after lifting
