# Supplementary material for: Characteristics and Related Factors of One-year Transition in Exercise Tolerance Following an Emergency Declaration due to the Coronavirus Disease 2019 Pandemic in Patients on Phase III Cardiac Rehabilitation
Source: Phys Ther Res. 2023 Apr 27;26(2):50–7. doi: 10.1298/ptr.E10232 (PMC10445121; doi:10.1298/ptr.E10232)
Supplement: Appendix 1. — Clinical characteristics in 56 participants [file ptr-26-50-s01.pdf]

Appendix 1. Clinical characteristics in 56 participants

	Overall (n = 56)	
Age, years	75.5	(71.0–79.0)
Men, n (%)	37	(66.1)
Body weight (kg)	60.2	± 10.9
BMI	23.5	(21.2–25.3)
Disease, n (%)		
Myocardial infarction	13	(23.2)
Angina pectoris	14	(25.0)
Postcardiac surgery	7	(12.5)
Chronic heart failure	19	(33.9)
Aortic disease	0	(0.0)
Peripheral artery disease	1	(1.8)
Post TAVI	2	(3.6)
Comorbidity, n (%)		
Hypertension	49	(87.5)
Dyslipidemia	41	(73.2)
Former or current smoker	30	(53.6)
Diabetes mellitus	19	(33.9)
Cardiovascular disease	20	(35.7)

Chronic kidney disease	12	(21.4)
Orthopedic disorders	16	(28.6)
Malignant tumor	8	(14.3)
Cerebrovascular disease	4	(7.1)
Respiratory disease	1	(1.8)
LVEF, %	60.0	(49.0–65.0)
β-blocker medication, *n* (%)	41	(73.2)
Multiple comorbidities 3 or more, *n* (%)	37	(66.1)
Worker, *n* (%)	14	(25.0)
Living alone, *n* (%)	15	(26.8)
Walk Score, points	77.0	(65.5–86.0)
Number of CR during the emergency declaration, times	3.6	± 2.7

Continuous variables are expressed as means with standard deviation in parenthesis or medians with interquartile range in parenthesis.

BMI, body mass index; CR, cardiac rehabilitation; LVEF, left ventricular ejection fraction; TAVI, transcatheter aortic valve implantation.

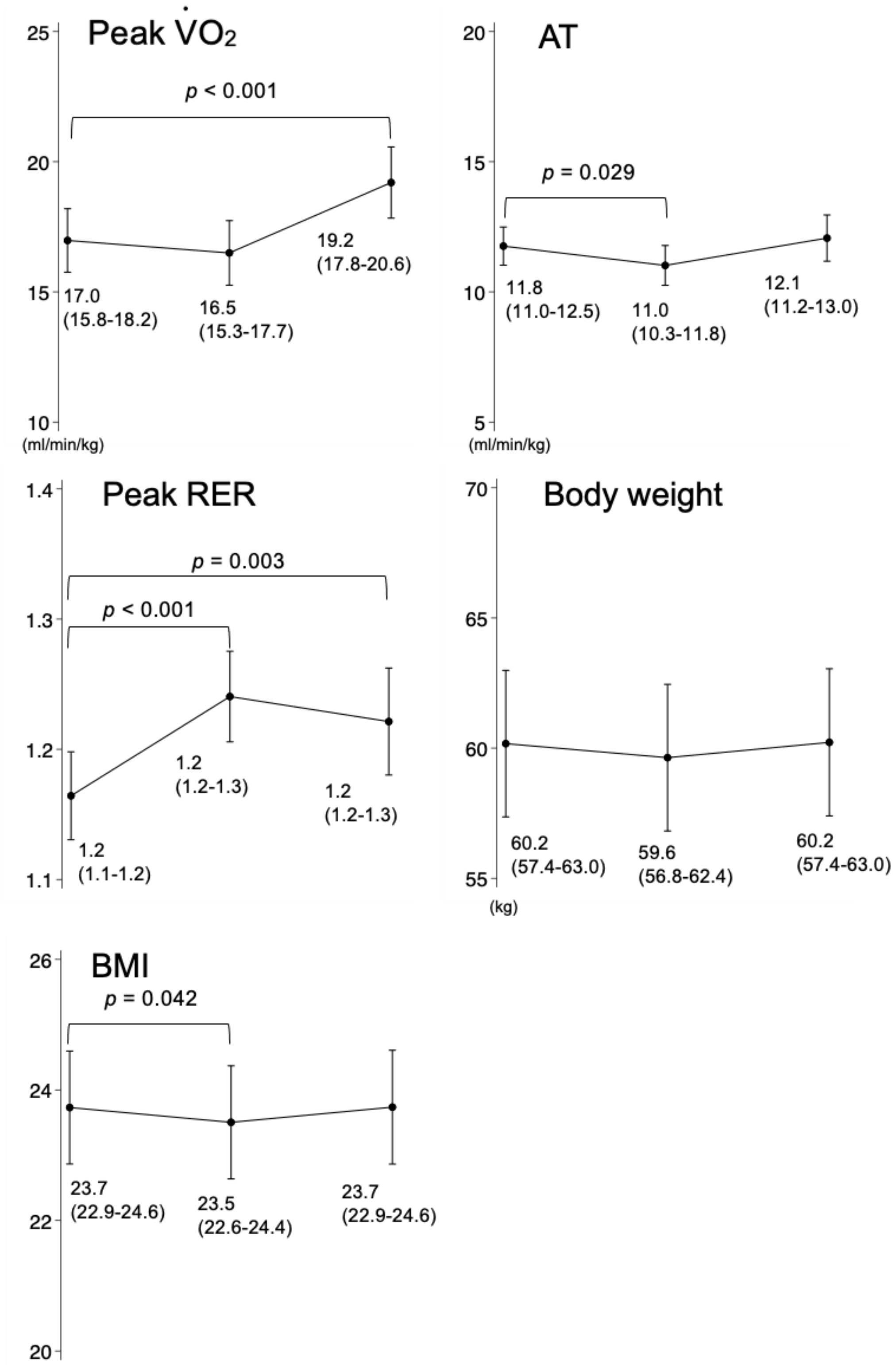
Peak $\dot{V}O_2$
p < 0.001
25
20
15
10
(ml/min/kg)
17.0
(15.8-18.2)
16.5
(15.3-17.7)
19.2
(17.8-20.6)
AT
p = 0.029
20
15
10
5
(ml/min/kg)
11.8
(11.0-12.5)
11.0
(10.3-11.8)
12.1
(11.2-13.0)
Peak RER
p = 0.003
p < 0.001
1.4
1.3
1.2
1.1
1.2
(1.1-1.2)
1.2
(1.2-1.3)
1.2
(1.2-1.3)
Body weight
70
65
60
55
(kg)
60.2
(57.4-63.0)
59.6
(56.8-62.4)
60.2
(57.4-63.0)
BMI
p = 0.042
26
24
22
20
23.7
(22.9-24.6)
23.5
(22.6-24.4)
23.7
(22.9-24.6)

Appendix 2.
